# Supplementary material for: Functional exploration of heterotrimeric kinesin-II in IFT and ciliary length control in Chlamydomonas
Source: eLife. 2020 Oct 28;9:e58868. doi: 10.7554/eLife.58868 (PMC7652414; doi:10.7554/eLife.58868)
Supplement: Figure 1—source data 1. [file elife-58868-fig1-data1.zip › Figure 1-Source Data 1/Figure1C_Movie_legend.docx]

**Figure 1C Movie legends**

Time-lapse movies of imaging microscopy of expressed wild-type CrKinesin-II and chimeric kinesin-II as indicated moving on microtubules *in vitro*. Images were acquired 12.3 pfs and the movies are played at the same speed. Bars, 2 μm.
