# Supplementary material for: Functional exploration of heterotrimeric kinesin-II in IFT and ciliary length control in Chlamydomonas
Source: eLife. 2020 Oct 28;9:e58868. doi: 10.7554/eLife.58868 (PMC7652414; doi:10.7554/eLife.58868)
Supplement: Figure 1—source data 2. [file elife-58868-fig1-data2.zip › Figure 1-Source Data 2/Figure1E_Movie_legend.docx]

**Figure 1E Movie legends**

Time-lapse movies of IFT46-YFP in flagella of *Chlamydomonas* cells expressing wild-type CrKinesin-II and chimeric CrKinesin-II. Images were acquired at 20 fps and movies are played at the same speed. Bars, 2 μm.
