# Supplementary figures and images for: Functional exploration of heterotrimeric kinesin-II in IFT and ciliary length control in Chlamydomonas
Source: eLife. 2020 Oct 28;9:e58868. doi: 10.7554/eLife.58868 (PMC7652414; doi:10.7554/eLife.58868)

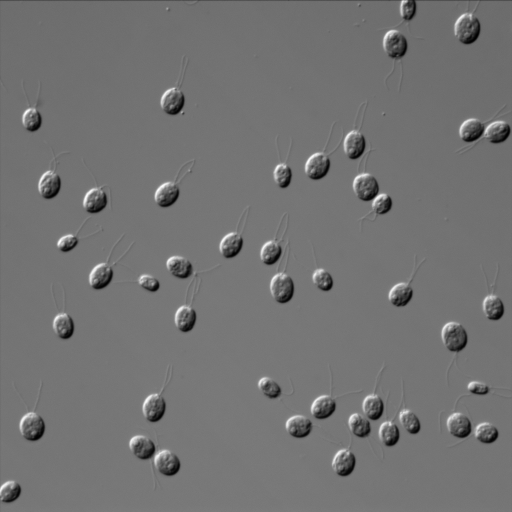

Supplement: Figure 2—source data 2. [file elife-58868-fig2-data2.zip › Figure 2-Source Data 2/Figure 2D-Representative cell images/7#.tif]

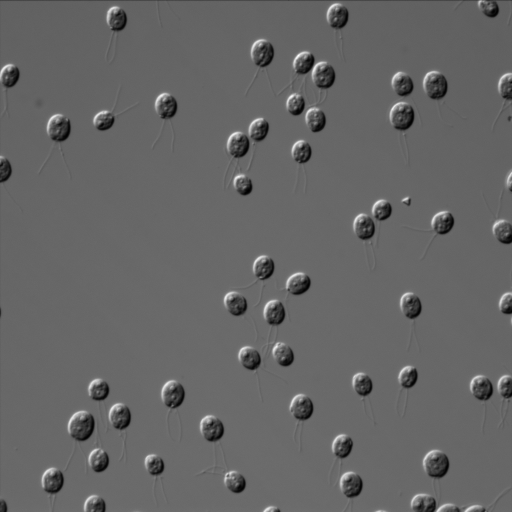

Supplement: Figure 2—source data 2. [file elife-58868-fig2-data2.zip › Figure 2-Source Data 2/Figure 2D-Representative cell images/8H2#.tif]

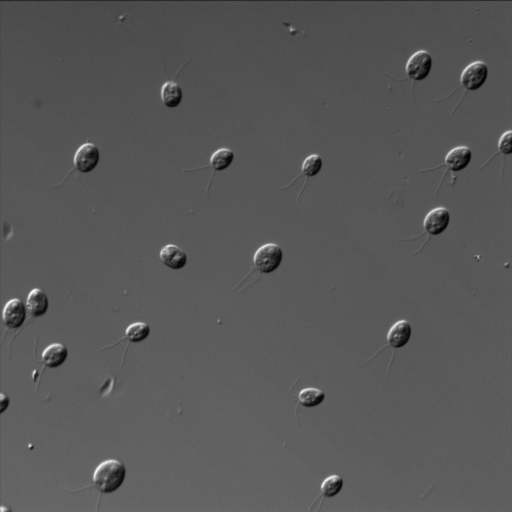

Supplement: Figure 2—source data 2. [file elife-58868-fig2-data2.zip › Figure 2-Source Data 2/Figure 2D-Representative cell images/RH19#.tif]

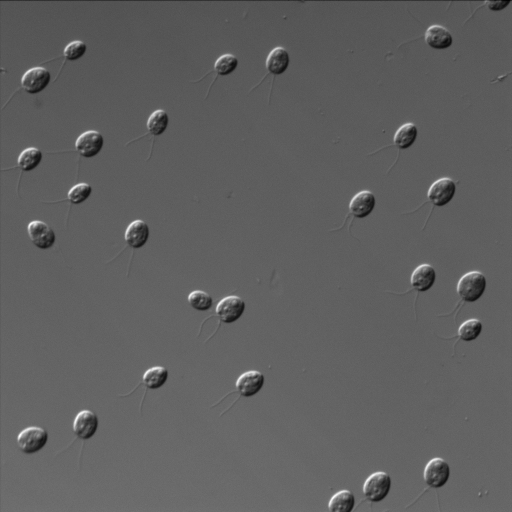

Supplement: Figure 2—source data 2. [file elife-58868-fig2-data2.zip › Figure 2-Source Data 2/Figure 2D-Representative cell images/RH4#.tif]

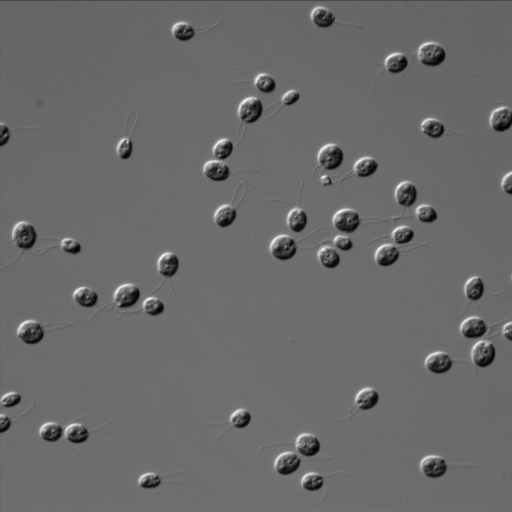

Supplement: Figure 2—source data 2. [file elife-58868-fig2-data2.zip › Figure 2-Source Data 2/Figure 2D-Representative cell images/RY1#.tif]

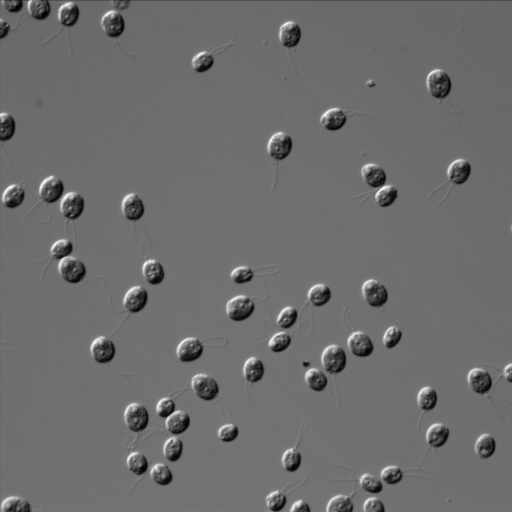

Supplement: Figure 2—source data 2. [file elife-58868-fig2-data2.zip › Figure 2-Source Data 2/Figure 2D-Representative cell images/RY5#.tif]

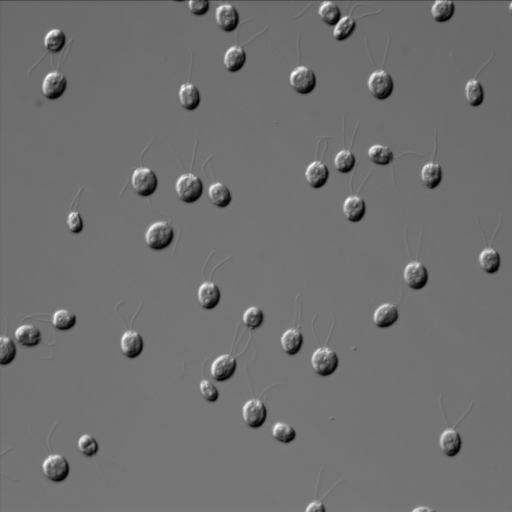

Supplement: Figure 2—source data 2. [file elife-58868-fig2-data2.zip › Figure 2-Source Data 2/Figure 2D-Representative cell images/WT.tif]

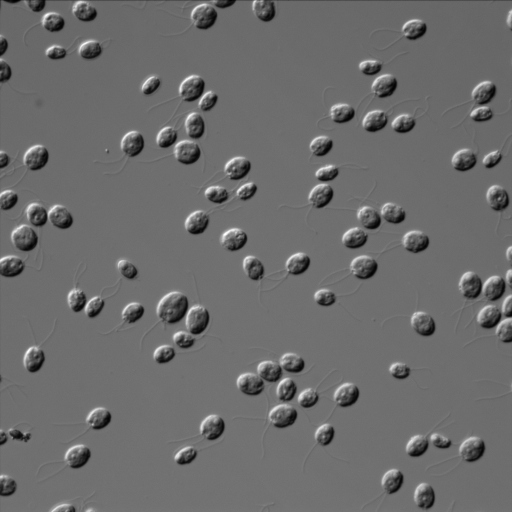

Supplement: Figure 4—source data 1. [file elife-58868-fig4-data1.zip › Figure 4-Source Data 1/Figure 4C_Representative images of cells/FLA8.FLA10.KAP.tif]

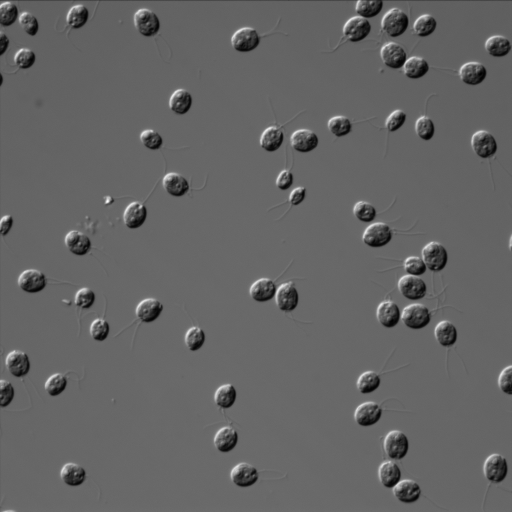

Supplement: Figure 4—source data 1. [file elife-58868-fig4-data1.zip › Figure 4-Source Data 1/Figure 4C_Representative images of cells/KIF3B'.FLA10.KAP.tif]

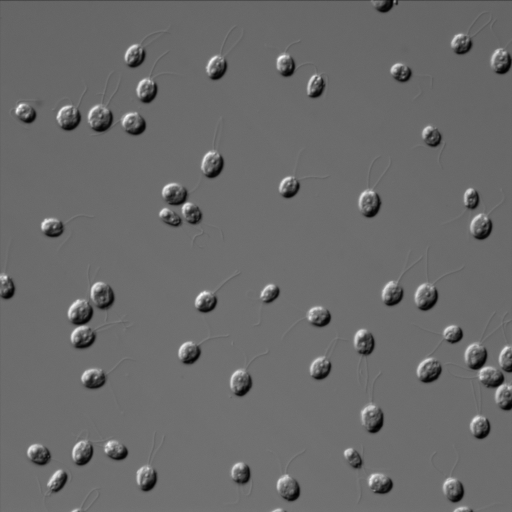

Supplement: Figure 4—source data 1. [file elife-58868-fig4-data1.zip › Figure 4-Source Data 1/Figure 4C_Representative images of cells/WT.tif]
