# Supplementary material for: Functional exploration of heterotrimeric kinesin-II in IFT and ciliary length control in Chlamydomonas
Source: eLife. 2020 Oct 28;9:e58868. doi: 10.7554/eLife.58868 (PMC7652414; doi:10.7554/eLife.58868)
Supplement: Figure 3—source data 1. [file elife-58868-fig3-data1.zip › Figure 3-Source Data 1/Figure 3A_Movie_legend.docx]

**Figure 3A Movie legends**

Time-lapse movies of FLA8-YFP or KIF3B’-YFP in *Chlamydomonas* cilia. FLA8-YFP or KIF3B’-YFP were expressed in *fla8* cells, respectively. Images were acquired at 20 fps, movies are played at the same speed. Bars, 2 μm.
