# Supplementary material for: Functional exploration of heterotrimeric kinesin-II in IFT and ciliary length control in Chlamydomonas
Source: eLife. 2020 Oct 28;9:e58868. doi: 10.7554/eLife.58868 (PMC7652414; doi:10.7554/eLife.58868)
Supplement: Figure 3—source data 2. [file elife-58868-fig3-data2.zip › Figure 3-Source Data 2/Figure 3B_C_F_Movie_legend.docx]

**Figure 3B_C_F Movie legends**

Time-lapse movies of IFT46-YFP of *Chlamydomonas* cilia of control cells, *fla8* cells expressing FLA8 or KIF3B’ or KIF3B’-YFP. Images were acquired at 20 fps, movies are played at the same speed. Bars, 2 μm. The data for anterograde velocity, IFT injection rate and IFT frequency are presented in Figure 3B, C and F, respectively.
